# Social representations as an epistemological matrix of health care and Nursing^*^

**DOI:** 10.1590/1518-8345.8047.4976

**Published:** 2026-10-23

**Authors:** Lise Maria Carvalho Mendes, Nayara Gonçalves Barbosa, Thais de Oliveira Gozzo, Diene Monique Carlos, Ana Karina Bezerra Pinheiro, Flávia Azevedo Gomes-Sponholz

**Affiliations:** 1 Universidade de São Paulo, Escola de Enfermagem de Ribeirão Preto, PAHO/WHO Collaborating Centre for Nursing Research Development, Ribeirão Preto, SP, Brazil.; 2 Universidade Federal do Amapá, Departamento de Ciências Biológicas e Saúde, Macapá, AP, Brazil.; 3 Universidade de São Paulo, Escola de Enfermagem, São Paulo, SP, Brazil.; 4 Universidade Federal do Ceará, Fortaleza, CE, Brazil.; 5 Scholarship holder at the Conselho Nacional de Desenvolvimento Científico e Tecnológico (CNPq), Brazil.

**Keywords:** Social Representation, Knowledge, Humanization of Assistance, Theoretical Models, Psychological Models, Social Psychology.

## Abstract

**Objective:**

to reflect on the epistemological aspects of Social Representations Theory in the field of health care and Nursing

**Method:**

this is a theoretical-reflective essay based on the conceptual reading of the main texts that influenced this theory. These texts were articulated with the literature in the health field.

**Results:**

social representations are the ways in which groups interpret the reality in which they live and guide their behaviors and practices. They function as a symbolic substance situated between what can be observed and what cannot be directly apprehended. From this perspective, they make it possible to understand how individuals and groups formulate their beliefs based on their experiences and on the values attributed to their lived experiences. In studies on health care and Nursing, this theory seeks to understand the representations established about care, as well as the meanings attributed to it.

**Conclusion:**

social representations bring researchers closer to the contexts that sustain meanings, allowing them to understand people’s beliefs, attitudes, and behaviors related to health and illness processes, thus supporting the development of therapeutic plans that are more aligned with the needs of those receiving care.

## Highlights

**(1)** Social Representations Theory allows care to be understood beyond its technical dimension.

**(2)** Knowledge does not circulate in a neutral manner.

**(3)** The naturalization of inequalities in institutionalized care is a challenge that must be overcome.

**(4)** The rigorous use of Social Representations Theory strengthens situated analyses and avoids universalizing interpretations.

## Introduction

Social Representations Theory (SRT), proposed by the Romanian scholar Serge Moscovici in the 1960s^1^, is situated within the realm of what is apprehended and externalized through speech, but also of what remains unsaid and emerges in everyday practice, behavior, repetition, collectivity, and the individual. When an individual attempts to deviate from the predominant practices of the collective, this deviation signals an exception and, therefore, confirms the existence of a certain rule. 

SRT highlights how people share and construct common meanings about objects, events, and social phenomena through processes of communication and social interaction. These representations shape the way we perceive the world around us and influence our beliefs, attitudes, and behaviors. This dialectical relationship, established between individual and social aspects, constitutes the theoretical basis of the concept of SRT, which seeks to understand the appropriation of social reality by the individual, or the manner in which the social is internalized^1^. 

To provide an epistemic contribution regarding social representations applied to studies of health care, it is necessary to articulate the concept of SRT within what is currently conceived as Science. This exercise may serve as a guiding element for understanding the advances that studies grounded in this framework may achieve. 

Although SRT has been widely mobilized in health and Nursing research, with relevant contributions already systematized in the literature, it is observed that a large portion of these studies has privileged its empirical or methodological applicability^2^ ^-^ ^3^. Over time, the recurrence of such studies has consolidated SRT as a fruitful framework for understanding the meanings attributed to care, without, however, exhausting the need for reflection on its epistemological foundations. 

In this context, the motivation for this essay arises from the need to critically revisit the epistemological assumptions of SRT, especially in light of its increasing, and at times uncritical, incorporation into the field of health and Nursing. Rather than merely reiterating its already recognized contributions, this manuscript proposes a reflective shift by questioning the ways in which SRT has been appropriated and the limits of its application when dissociated from its original theoretical matrix, with the aim of qualifying its critical and contextualized use in the present. 

Thus, this article aims to reflect on the epistemological aspects of Social Representations Theory in the field of health care and Nursing.

## Method

### Study design 

This is a theoretical-reflective essay^4^, understood as a modality of scientific production aimed at the critical, interpretative, and argumentative analysis of concepts, theories, and epistemological assumptions, without the intention of empirical exhaustion or exhaustive systematization of the literature. This type of study privileges reflective exercise grounded in consistent theoretical frameworks, articulating conceptual trajectories, tensions, and interpretative limits based on the authors’ academic experience and on critical dialogue with classical and contemporary works^4^, with the aim of reflecting on the epistemological aspects of SRT^1^ in the field of health care and Nursing. 

### Sample definition

A survey of the foundational works of SRT was conducted, with emphasis on the writings of Serge Moscovici, which delineated the foundations of this theory within the field of social psychology. Reference texts by other classical authors, such as Durkheim and Freud, as well as works by contemporary authors, such as Pierre Bourdieu, were included in order to understand the theoretical and contextual reflections involved in the formulation of SRT. 

The texts were selected based on their relevance to the epistemological analysis of the theory and to its application in the field of health care. In addition to the main works, studies exploring related concepts were also included, such as those examining the relationship between social representations and cultural and health practices.

### Period

The temporal scope of the survey and reading process focused on the period from January to April 2024, without excluding the incorporation of timeless classical works. 

### Data processing and analysis

The theoretical reflection was conducted through a comprehensive and interpretative analysis of the selected texts^5^, guided by assumptions of socio-historical and epistemological analysis. The reading of the texts followed a critical and reflective perspective, prioritizing: (i) the understanding of the epistemological foundations of the works; (ii) the identification of convergences, tensions, and conceptual shifts among the authors; and (iii) the articulation of these concepts with the empirical and theoretical field of health care and Nursing. 

Throughout the process, three meetings were held among the authors to discuss the texts and to construct analytical consensus. These collective discussions guided both the organization of the reflections and the argumentative structure of the essay, enabling conceptual refinement and internal coherence of the manuscript. Based on this analytical process, a reflective axis guiding the presentation of the results was organized, entitled “A look at social representations”, constructed as an interpretative synthesis of the conceptual foundations mobilized by the analyzed authors.

### Ethical aspects

This study is a theoretical essay, based exclusively on publicly available secondary sources, with no involvement of human subjects or collection of primary data. Therefore, it does not fall under the provisions of Brazilian National Health Council (CNS, in Portuguese) Resolutions nº 466/2012 and nº 510/2016, and submission to a Research Ethics Committee was not required.

## Results

Initially, it is important to highlight the influence of the French sociologist Émile Durkheim, considered a precursor of social representations, who, in his reflections, provided important contributions to the study of collective representations^6^. The author emphasized the importance of collective representations in the cohesion and maintenance of social order. He argued that the beliefs, values, and norms shared by members of a society form a collective consciousness that shapes individual behavior and promotes social cohesion^6^. 

Within the historical development of the Sciences, the theoretical-clinical framework of Sigmund Freud also exerted an influence on Moscovici. Moscovici drew on this influence to formulate an understanding of the gaps in the manifestations of consciousness, suggesting the presence of another order^7^. For Freud, the unconscious should not be understood as a manifestation of irrationality nor as a hidden consciousness. On the contrary, he considered it a legitimate and essential hypothesis, without which phenomena related to the functioning of the psychic apparatus would continue to appear incoherent and incomprehensible to Science^7^. For Moscovici, this order would assume a prominent role and would be seen as endowed with its own activity^1^. 

Thus, in dealing with the truth of its object, psychoanalysis produced a significant shift in relation to modern philosophy by proposing the decentralization of reason and by placing consciousness under suspicion, attesting to the dimension of subjectivity while simultaneously affirming the existence of the unknown and the not-yet-known^7^.

In a perspective diametrically opposed to that of the unconscious, Durkheim’s reflections^6^ indicate that social facts are external to the individual and exist independently of our awareness of them. According to this author, human beings come into contact with these facts from birth and internalize many of them. Moscovici was influenced by Durkheim in the construction of SRT. For both authors, mental practices originate in society and therefore constitute social categories of thought^1^ ^,^ ^6^. 

However, it should be noted that, within sociology, there are several ways of systematizing the understanding of representations. Post-war authors share a convergent point in their works: the overcoming of the dichotomy between subjectivity and objectivity in the relationship between the individual and society. Sociologists contemporary to Moscovici, such as the French sociologist Pierre Bourdieu, emphasized from the beginning of their academic trajectories the importance of the symbolic dimension in the construction of social reality, seeking to understand how individuals construct meanings within social contexts^8^. 

Bourdieu concluded that representations materialize in social practices and institutions, although they are not independent from them. For this author, the object must be understood from a relational perspective, in which each element is defined by the relations that connect it to others within a vast network of systems. Within this network, elements acquire their meanings and functions in society^8^. The differences between the theoretical postulates of Moscovici and Bourdieu regarding representations lie in the emphasis given to symbolic constructions. Moscovici gives greater prominence to the structuring structure, whereas Bourdieu highlights the structured structure^1^ ^,^ ^8^. In other words, Moscovici focuses on the process of constructing structures, while Bourdieu emphasizes already established structures.

Moscovici’s generation intensely debated how Science influenced historical transformation, modes of thought, and social perspectives, while also analyzing how these ideas could be integrated into people’s beliefs^1^. A belief can be understood as an idea sustained by a specific cultural group, serving as the basis for evaluations that lead to the acceptance of something as true even without empirical evidence. In this sense, a representation consists of an organized set of such beliefs, which guides a group of people in relation to an object within the social space^1^. 

The distinction between belief and knowledge lies in the fact that scientific knowledge, theoretically, can be demonstrated through empirical evidence. Moscovici questioned how scientific knowledge becomes transformed into belief and “spontaneous” knowledge^1^. The author sought to overcome the scientific model that attributed excessive importance to scientific knowledge to the detriment of the culture of common sense, which is constructed and shaped through everyday communication and itself constitutes a form of culture. 

Moscovici’s thesis addresses the knowledge of French society about psychoanalysis, and its conclusions can be summarized in three fundamental aspects. First, social representations function as an intermediate point between the understanding of psychoanalysis within French society and scientific knowledge. Second, representations do not hold the same meaning for all members of society, since they depend on individuals’ sociocultural contexts. Third, when facing new situations or new objects, such as psychoanalysis, the process of representation follows a logical sequence: unfamiliar objects become familiar through a dual mechanism known as anchoring and objectification. This process allows individuals or groups to connect real, concrete, and comprehensible images drawn from their everyday lives to new objects they encounter and need to understand^1^.

The processes of anchoring and objectification are essential for understanding how social representations are formulated, shared, and transmitted within a group, influencing people’s perceptions and responses to the world around them. The concept of anchoring refers to the process through which new information is related to concepts or ideas already existing in people’s minds. For example, when individuals become aware of a new medical therapy, they may anchor this information in their previous beliefs about health, medicine, and treatments. It is the process through which what is unfamiliar is incorporated into a preexisting system of social thought, allowing older representations to accommodate new ones so that the unfamiliar becomes familiar^1^.

The concept of objectification, in turn, refers to the process through which social representations are expressed and manifested in concrete objects, practices, or symbols. In this way, social representations become visible and tangible in everyday life through the process of objectification^1^. This may include rituals, language, cultural artifacts, and institutions that reflect and reinforce the beliefs and values shared within a social group. For example, symbols in the field of health, such as white coats, stethoscopes, and shamans, may represent a community’s beliefs and values regarding health treatment. The objectification of a concept or object also depends on sociocultural factors, such as differential access to information according to individuals’ social positions and the values and norms of the group to which they belong.

Social representations are allegorical in nature, situated between what can be observed and what cannot be directly grasped. Thus, social representations can be understood as concrete realities, although not necessarily tangible or measurable. They may be compared to the notion of charisma: something imprecise but that, when present in certain individuals, exerts a real and concrete influence^1^. Charisma is a concrete manifestation, yet practically intangible. Social representations constitute this symbolic substance that blends with ideas and beliefs, as occurs in religions, myths, and science. They are specific phenomena linked to a particular way of understanding and communicating, fundamental to the constitution of reality and common sense, forming part of the symbolic fabric of social life.

## Discussion

Contemporary notions of health and Nursing are not constituted exclusively through technical-scientific advances but are also shaped by historically produced social representations that guide ways of thinking, evaluating, and practicing care. In this sense, the forms of knowledge that underpin certain epistemologies in health express beliefs shared within specific sociocultural contexts^9^. 

According to SRT, such knowledge does not circulate in a neutral manner but is anchored in preexisting symbolic systems and objectified in practices, institutional discourses, and existing models of care^1^. Thus, what is recognized as valid knowledge in health and Nursing reflects not only scientific criteria but also social representations characteristic of a particular historical moment, which influence the definitions of normality, risk, and care^9^.

 An example of this can be observed in the social representations surrounding the body, health, illness, and risk, which, in different historical contexts, define what is considered normal or pathological^9^. As scientific knowledge circulates socially, it is appropriated, reinterpreted, and transformed into forms of common-sense knowledge, thereby guiding institutional and professional practices^1^ ^,^ ^10^. In the field of health, this process becomes evident when certain models of care, such as the hospital-centered model or the biomedical model, are consolidated not only because of their technical effectiveness but also because they are anchored in social representations that associate care with specialized intervention, medicalization, and the centrality of professional expertise^11^ ^-^ ^12^.

From this perspective, epistemologies in health and Nursing are constructed in continuous dialogue with the symbolic systems of their time, expressing socially shared values and beliefs. In this field, social representations play an important role in how individuals perceive, express, and respond to care. They incorporate beliefs, values, stereotypes, attitudes, and behaviors shared within a specific community or social group^1^. These representations may influence several aspects of health care, such as the search for treatment, adherence to prescribed therapies, perceptions of the risk of contracting diseases, attitudes toward health professionals, and health-related lifestyle choices. 

The major paradox in the study of representations within the field of health lies in attempting to explain a phenomenon while acknowledging the impossibility of fully grasping the essence of things^12^. It should be emphasized that illness is not an experience limited solely to biological alteration; rather, it also constitutes a substrate of sociocultural construction. The notion of illness depends on the conception of what it represents or means to human beings^11^. Emphasizing illness as the result of a combination of aspects of individual experience and sociocultural contexts does not imply considering it merely a social construction while disregarding biological aspects. On the contrary, sociocultural discussion must be articulated with the biological dimensions of illness in order to promote an interdisciplinary and integrated perspective in approaching this phenomenon.

Even from a formal standpoint, the relationship between health services and users involves many aspects beyond the physical encounter between professionals and patients in the settings where care is provided. Among these factors are local health policies and individuals’ conceptions of what it means to be ill and what it socially represents to seek professional assistance. In this way, within every culture and society, illness, as well as the responses to it, the individuals who experience it, those who undertake its treatment, and the institutions involved, are interconnected within a broad network of meanings and representations^12^. 

The components of this system include, among other elements, beliefs about the origins of diseases, the ways in which treatment is sought and evaluated, the roles performed, and the power relations among all actors involved^12^. For instance, a specific cold may be caused by the same infectious agent anywhere in the world, producing similar pathophysiological changes in different individuals. However, the available health system, the treatments offered, and the perception that each affected individual develops regarding their own health may manifest in very different ways.

In this regard, the concept of symbolic efficacy is mobilized to characterize the effects obtained through the involvement of beliefs and their symbolic dimensions in the physical-organic world, more specifically, within the human organism, thereby blurring the boundary between the conscious and the unconscious. In the modern world, healing often derives from the ingestion of a biochemical compound intended to produce the expected organic response. What is often overlooked, however, is that belief may act from the unconscious toward the conscious, also producing changes in the organism’s *status quo* ^11^. 

Thus, the interpretation of illness shifts toward the realm of intersubjectivity, everyday experience, and the ways in which individuals express, interpret, and communicate their experiences. The coping with suffering is also connected to social structures rather than solely to the individual. Therefore, suffering related to illness can be understood as a collective social construction of reality, frequently influenced by historical-cultural, ideological, and sociopolitical contexts associated with illness.

To illustrate this reflection, one may consider the role of a shaman in a difficult childbirth experienced by an Indigenous woman and the implications of musical and healing rituals in shaping how this woman interprets and attributes meaning to the pain associated with the childbirth process^13^. From this perspective, such reflections may provide clues to understanding the healing power of a placebo, as well as the role of social representations anchored in information conveyed by health professionals, which may ultimately influence morbidity and mortality indicators. An example of this is the high number of elective cesarean sections performed in Brazil, often without clinical indication and, in such cases, without scientific advantages capable of legitimizing them. 

From this perspective, certain social representations may guide institutional practices that naturalize inequalities, legitimize inadequate conduct, or sustain interventions dissociated from the best available scientific knowledge, producing concrete effects and, at times, serious social harm. Studies grounded in SRT show that professional practices are not neutral but are permeated by beliefs, attitudes, and behaviors shared within institutional fields, which guide clinical and organizational decisions, even in contexts characterized by high levels of technical regulation. 

Research conducted with Nursing professionals demonstrates that certain representations structure ways of acting and justifying care practices, even when these are in tension with well-established scientific guidelines^10^ ^,^ ^14^. Situations such as institutional racism, the delegitimization of the suffering of certain groups, or adherence to ideologically oriented therapeutic models, such as those observed during the COVID-19 pandemic with the prescription of medications lacking scientific evidence^14^, illustrate how social representations, when incorporated into professional practices, may compromise the quality of care, challenge ethical principles, and negatively impact health outcomes.

Among studies on social representations are those that seek to understand the origin of specific representations, identifying them through language, that is, through speech and human behavior, whether individual or collective. In this regard, a study^15^ conducted in the French village of Ainay-le-Château, where a process of deinstitutionalization of individuals experiencing psychological suffering was underway, sought to understand how madness was represented. The deinstitutionalization process encouraged coexistence between local residents and individuals experiencing mental suffering through a hosting system. The guiding question of the study was: “How do social representations of madness explain the relationship with the mentally ill person as a figure of otherness?” 

The study concluded, among other findings, that residents predominantly associated madness with fear of contagion. This perception led them to adopt behaviors inconsistent with the scientific knowledge disseminated at the time. Although residents claimed in their discourse that psychological suffering was not transmissible through the sharing of household utensils and clothing, their actions reflected the opposite.

Thus, social representations contained non-verbalized aspects that materialized in the everyday behavior of the villagers, such as washing separately the clothes and utensils of people experiencing psychological suffering. When questioned about these practices, residents responded that it was a matter of “custom”, inherited from their parents and grandparents due to fear of contagion. In this way, although they verbally denied the possibility of transmission, they maintained in practice a strict separation of washing water in everyday domestic life, even in the face of the apparent “irrationality” of such behavior^15^. 

As a social practice, studies within this field of knowledge have addressed different health needs, drawing on SRT and enabling advances across diverse themes and contexts of care. The incorporation of SRT marked an epistemological turning point in Nursing scientific production by enabling the overcoming of strictly positivist approaches and expanding the understanding of care as a social and symbolic practice^2^. SRT shows affinity with the objects of Nursing, which are characterized by the refusal to hierarchize forms of knowledge and by valuing the intersections between scientific knowledge, common sense, and beliefs^3^. The analysis of the trajectory of research in Nursing reveals that this framework has been particularly fruitful in interpreting complex phenomena of the health-illness process, care practices, and professional identity, allowing a better understanding of how beliefs, attitudes, and behaviors become stabilized and transformed in everyday institutional life. 

A study that analyzed nurses’ social representations regarding the care provided to women in situations of sexual violence revealed that such representations are anchored in the implementation of protocols carried out in a humanized manner, with objectification expressed through welcoming and supportive care practices^16^. Another study showed that, for nurses, the quality of care in hospital settings is strongly influenced by structural limitations, such as overcrowding and scarcity of material resources^17^. 

It can be observed that social representations influence forms of interaction with the social environment. Social research approaches grounded in SRT and applied to Nursing aim to explore how care is perceived and to understand the meanings attributed to it. This involves analyzing the contexts that shape these meanings, allowing for a better understanding of people’s behaviors, attitudes, and beliefs regarding health and illness. These approaches also contribute to the formulation of therapeutic plans more closely aligned with the needs of those receiving care^18^.

In this context, it is important to analyze the personal beliefs involved in the interaction between the individual who receives health care and the system that provides it, in order to support improvements in policies, programs, services, and the training of health professionals. Understanding how users perceive health and illness processes is fundamental for improving the quality of services, increasing both access to and the effectiveness of care. In this way, it becomes necessary to understand users’ opinions and behaviors when facing illness. Such knowledge may reveal gaps in services as perceived by users themselves, influencing how similar groups respond to treatments and preventive guidance. Therefore, research on representations related to health care is essential for guiding the formulation of more appropriate health policies.

However, it should be noted that attempts to define the concept of social representations too rigidly carry the risk of transforming what should be interpreted as situational phenomena into permanent ones. Examples include studies that seek to represent “the thinking of young people”, “the thinking of women”, or the “worldviews of poor people”, highlighting the need for caution when employing this concept. It is important to recognize that opinions shared by informants often emerge from heterogeneous cultural contexts, which cannot always be unified through surveys or questionnaires^19^.

A competent observer is one who not only observes others but also observes themselves, promoting a conscious interaction that emphasizes otherness. Knowledge is constructed through the confrontation of different cultural positions present within this interaction^8^. The researcher is not neutral while listening and observing; rather, they attempt to understand the perspective of the observed and confront it with their own. Moreover, it is important to recognize that the investigator, in the role of interpreter, becomes their own reader, and the observer becomes a product of their own reflection^5^.

At times, positivism and hypothetico-deductive logic erroneously suggest that there exists a neutral rationality and that Science can develop without being influenced by the social representations of a given context^1^. As an illustration, when reflecting on the urgency of eliminating maternal deaths from preventable causes, one might question whether such efforts are also anchored in social representations intertwined with philosophical conceptions of death and social conceptions of motherhood, structured by historical, political, and economic convergences that shape Science as conceived within a specific time and space. 

Directing attention to one’s own standpoint in relation to the object, and to everything that results from this relationship, is a *sine qua non* condition^8^. In this sense, it is suggested that the previously assumed and socially recognized position of the objective and objectifying observer, who imposes their own norms of construction upon the object, should be abandoned. 

The challenge lies in avoiding the structural realism to which objectivism inevitably leads, a tendency that attributes absolute reality to something that is, in fact, relative - a form of hypostatization. This occurs when structures are treated as pre-established realities independent of the history of the individuals and groups who share them. At the same time, it is essential not to fall into subjectivism, which proves incapable of adequately addressing the complexities of the social world. To overcome this dichotomy, it is necessary to return to practice, understood as the space of dialectics where incorporated historical products, macrostructures, and fields of power intersect^8^.

Situating the context of social representations and seeking to understand their mechanisms of construction may constitute a valuable pathway for analyzing the values and meanings produced by specific social groups. In line with this reflection, some guiding questions can help avoid such analytical pitfalls: Who knows and from where do they know? What do they know and how do they know it? What do they know about and with what effects? These questions refer to the conditions of production and circulation of social representations, the processes and states of social representations, and the epistemological status of social representations^15^. 

Socio-historical analysis requires from the researcher a critical stance capable of encompassing the social structure, understood as a space marked by asymmetries and relatively stable differences that characterize social institutions and fields of interaction within social structures. To illustrate this premise, one may consider the differences between narratives about illness produced by women and men, which reflect the gender roles performed within the groups analyzed. 

The experience of being ill may share common aspects among individuals regardless of biological sex. However, the roles performed and the meanings attributed to this condition may vary significantly, assuming particular characteristics according to the cultural constructions of gender. In some regions of the world, cultural differences and the social roles performed by men and women are so pronounced that, although they coexist under the same national framework, these groups experience realities almost as if they belonged to distinct universes. Thus, social groups construct their own representations by identifying, interpreting, accepting, or rejecting certain values and ways of living, thereby producing their own systems of meaning^20^.

This essay contributes to the advancement of scientific knowledge by critically revisiting the epistemological foundations of SRT within the fields of health and Nursing. In doing so, it shifts the predominant focus of the literature, largely centered on empirical applicability, toward the problematization of its theoretical assumptions and conditions of use. The study highlights how social representations operate not only in the reception of care practices but also in the construction of knowledge itself, models of care, and professional practices in health. In this way, the manuscript provides support for a more rigorous, critical, and contextualized use of SRT in future research, contributing to strengthening its theoretical incorporation within the fields of public health and Nursing.

As limitations, it should be noted that, as a theoretical-reflective essay, the study does not propose to conduct empirical analysis or a systematic review of the literature, which restricts the possibility of generalizations or direct comparisons among different contexts in which SRT is applied. Furthermore, the selection of works prioritized classical texts and reference authors. Although this approach is coherent with the epistemological objective of the reflection, it may not encompass the entirety of contemporary productions that employ the theory in specific empirical approaches in a systematic manner. Nevertheless, these limitations do not compromise the validity of the proposal, since the aim was to deepen the theoretical and epistemological reflection, rather than to exhaust the debate or map the existing body of research.

## Conclusion

SRT, when applied to health care and Nursing, contributes to illuminating the beliefs, attitudes, and behaviors that influence the provision of care, enabling a more effective and integrative approach to caring for individuals. Understanding social representations in the field of health is essential for the development of interventions and for strengthening more effective care practices. This involves recognizing and respecting the beliefs and values of local communities, as well as engaging in dialogue with their members during the development of culturally sensitive health programs. 

However, the application of SRT also presents epistemological challenges that deserve explicit consideration. It is frequently observed that insufficient epistemological grounding becomes a barrier to moving beyond the level of common sense in research on social representations related to health care and Nursing. It is also important to highlight the risk of reifying social representations when they are interpreted as permanent and homogeneous structures, disregarding their dynamic and relational nature. Such interpretations may lead to undue generalizations and to the oversimplification of contexts marked by social, historical, and cultural heterogeneities. 

## Data Availability

All data generated or analysed during this study are included in this published article.
