# Supplementary material for: Social representations as an epistemological matrix of health care and Nursing
Source: Rev Lat Am Enfermagem. 2026 Oct 23;34:e4976. doi: 10.1590/1518-8345.8047.4976 (PMC13706265; doi:10.1590/1518-8345.8047.4976)
Supplement: Translation

# Translation: Representações sociais como matriz epistemológica do cuidado em saúde e Enfermagem^*^

**DOI:** 10.1590/1518-8345.8047.4977

**Authors:** Lise Maria Carvalho Mendes, Nayara Gonçalves Barbosa, Thais de Oliveira Gozzo, Diene Monique Carlos, Ana Karina Bezerra Pinheiro, Flávia Azevedo Gomes-Sponholz

**Affiliations:** 1 Universidade de São Paulo, Escola de Enfermagem de Ribeirão Preto, PAHO/WHO Collaborating Centre for Nursing Research Development, Ribeirão Preto, SP, Brasil.; 2 Universidade Federal do Amapá, Departamento de Ciências Biológicas e Saúde, Macapá, AP, Brasil.; 3 Universidade de São Paulo, Escola de Enfermagem, São Paulo, SP, Brasil.; 4 Universidade Federal do Ceará, Fortaleza, CE, Brasil.; 5 Scholarship holder at the Conselho Nacional de Desenvolvimento Científico e Tecnológico (CNPq), Brasil.

**Keywords:** Representação Social, Conhecimento, Humanização da Assistência, Modelos Teóricos, Modelos Psicológicos, Psicologia Social.

## Abstract

**Objetivo:**

refletir sobre os aspectos epistemológicos da Teoria das Representações Sociais no campo do cuidado em saúde e Enfermagem.

**Método:**

trata-se de ensaio teórico-reflexivo, no qual foi utilizada, como base conceitual, a leitura dos principais textos que influenciaram essa teoria. Tais textos foram articulados à literatura da área da saúde.

**Resultados:**

as representações sociais são a forma como os grupos interpretam a realidade em que vivem e orientam suas condutas e práticas. Elas atuam como uma substância simbólica, situada entre aquilo que pode ser observado e aquilo que não pode ser atingido. Nessa perspectiva, tornam possível compreender como indivíduos e grupos formulam suas crenças com base em suas experiências e nos valores atribuídos às suas vivências. Nas pesquisas sobre cuidado em saúde e Enfermagem, essa teoria busca compreender as representações estabelecidas sobre o cuidado, bem como conhecer os significados a ele atribuídos.

**Conclusão:**

as representações sociais aproximam os pesquisadores dos contextos que sustentam os significados, permitindo compreender crenças, atitudes e comportamentos das pessoas relacionados aos processos de saúde e doença, e auxiliando a conduzir planos terapêuticos mais alinhados às necessidades do outro a quem o cuidado se destina.

## Destaques

**(1)** A Teoria das Representações Sociais permite compreender o cuidado para além de sua dimensão técnica.

**(2)** Os conhecimentos não circulam de forma neutra.

**(3)** A naturalização das desigualdades no cuidado institucionalizado é um desafio a ser superado.

**(4)** O uso rigoroso da Teoria das Representações Sociais qualifica análises situadas e evita leituras universalizantes.

## Introdução

A Teoria das Representações Sociais (TRS), proposta pelo romeno Serge Moscovici na década de 1960^1^, insere-se no universo do que é apreendido e exteriorizado pela fala, mas também do que não é dito e emerge na prática cotidiana, no comportamento, na repetição, na coletividade e no indivíduo. Este, ao tentar desviar da prática predominante da coletividade, assinala que é uma exceção e, portanto, confirma a existência de uma determinada regra. 

A TRS destaca como as pessoas compartilham e constroem significados comuns sobre objetos, eventos e fenômenos sociais por meio de processos de comunicação e interação social. Essas representações moldam a maneira como percebemos o mundo ao nosso redor e influenciam nossas crenças, atitudes e comportamentos. Essa relação dialética, estabelecida entre os aspectos individuais e sociais, constitui a base teórica do conceito da TRS, que busca compreender a apropriação da realidade social pelo indivíduo ou o modo como o social é internalizado^1^. 

Para uma contribuição epistêmica acerca das representações sociais aplicadas aos estudos de cuidado em saúde, faz-se necessário articular o conceito da TRS ao que hoje se concebe como Ciência. Acredita-se que esse exercício pode atuar como elemento condutor para a compreensão dos avanços que estudos apoiados nesse referencial podem vislumbrar. 

Embora a TRS venha sendo amplamente mobilizada nas pesquisas em saúde e Enfermagem, com contribuições relevantes já sistematizadas na literatura, observa-se que grande parte desses estudos tem privilegiado sua aplicabilidade empírica ou metodológica^2^ ^-^ ^3^. Ao longo do tempo, a recorrência dessas produções consolidou a TRS como referencial fecundo para a compreensão dos sentidos atribuídos ao cuidado, sem, contudo, esgotar a necessidade de reflexão sobre seus fundamentos epistemológicos. 

Nesse contexto, a motivação deste ensaio decorre da necessidade de retomar criticamente os pressupostos epistemológicos da TRS, especialmente frente à sua incorporação crescente e, por vezes, acrítica, no campo da saúde e Enfermagem. Mais do que reiterar suas contribuições já reconhecidas, este manuscrito propõe um deslocamento reflexivo, interrogando os modos pelos quais a TRS tem sido apropriada e os limites de sua aplicação quando dissociada de sua matriz teórica original, com vistas a qualificar seu uso crítico e contextualizado no presente. 

Dessa forma, este artigo tem como objetivo refletir sobre os aspectos epistemológicos da Teoria das Representações Sociais no campo do cuidado em saúde e Enfermagem.

## Método

### Delineamento do estudo 

Trata-se de um ensaio teórico-reflexivo^4^, compreendido como uma modalidade de produção científica voltada à análise crítica, interpretativa e argumentativa de conceitos, teorias e pressupostos epistemológicos, sem a pretensão de esgotamento empírico ou de sistematização exaustiva da literatura. Esse tipo de estudo privilegia o exercício reflexivo, fundamentado em referenciais teóricos consistentes, articulando trajetórias conceituais, tensões e limites interpretativos, a partir da experiência acadêmica dos autores e do diálogo crítico com obras clássicas e contemporâneas^4^, com o objetivo de refletir sobre os aspectos epistemológicos da TRS^1^ no campo do cuidado em saúde e Enfermagem. 

### Definição da amostra

Foi realizado um levantamento das obras fundadoras da TRS, com destaque para os trabalhos de Serge Moscovici, que delinearam os fundamentos dessa teoria no campo da psicologia social. Foram incluídos textos de referência de outros autores clássicos, como Durkheim e Freud, e de autores contemporâneos, como Pierre Bourdieu, para compreender as reflexões teóricas e contextuais envolvidas na formulação da TRS. 

Os textos foram selecionados com base em sua relevância para a análise epistemológica da teoria e para sua aplicação no campo do cuidado em saúde. Além das obras principais, foram incluídos estudos que exploram conceitos relacionados, como aqueles que examinam a relação entre representações sociais e práticas culturais e de saúde.

### Período

O recorte temporal do levantamento e da leitura concentrou-se no período de janeiro a abril de 2024, sem prejuízo da incorporação de obras clássicas atemporais. 

### Tratamento e análise de dados

A reflexão teórica foi conduzida por meio de uma análise compreensiva e interpretativa dos textos selecionados^5^, orientada por pressupostos da análise sócio-histórica e epistemológica. A leitura dos textos foi orientada por uma perspectiva crítica e reflexiva, priorizando: (i) a compreensão dos fundamentos epistemológicos das obras; (ii) a identificação de convergências, tensões e deslocamentos conceituais entre os autores; e (iii) a articulação dos conceitos com o campo empírico e teórico do cuidado em saúde e Enfermagem. 

Ao longo do processo, foram realizadas três reuniões entre os autores para debate dos textos lidos e construção de consensos analíticos. Essas discussões coletivas orientaram tanto a organização das reflexões quanto a estrutura argumentativa do ensaio, permitindo o refinamento conceitual e a coerência interna do texto. A partir desse percurso analítico, foi organizado o eixo reflexivo que orientou a apresentação dos resultados, denominado “Um olhar sobre as representações sociais”, construído como síntese interpretativa dos fundamentos conceituais mobilizados pelos autores analisados.

### Aspectos éticos

Trata-se de um ensaio teórico, fundamentado exclusivamente em fontes secundárias de domínio público, sem envolvimento de seres humanos ou coleta de dados primários, não se enquadrando nas disposições das Resoluções CNS nº 466/2012 e nº 510/2016, sendo dispensada a apreciação por Comitê de Ética em Pesquisa.

## Resultados

De início, é possível destacar a influência do sociólogo francês Émile Durkheim, considerado precursor das representações sociais, ao tecer, em suas reflexões, apontamentos para o estudo das representações coletivas^6^. O autor enfatizou a importância das representações coletivas na coesão e na manutenção da ordem social. Argumentou que as crenças, valores e normas compartilhados pelos membros de uma sociedade formam uma consciência coletiva que molda o comportamento individual e promove a coesão social^6^. 

No contexto do desenvolvimento histórico das Ciências, a estrutura teórico-clínica de Sigmund Freud também exerceu influência sobre Moscovici. Este utilizou essa influência para formular uma compreensão das lacunas nas manifestações da consciência, sugerindo a presença de uma outra ordem^7^. Para Freud, o inconsciente não deveria ser visto como uma manifestação da irracionalidade, nem como uma consciência oculta. Ao contrário, ele o considera uma hipótese legítima e essencial, sem a qual os fenômenos relacionados ao funcionamento do aparelho psíquico continuariam a parecer incoerentes e incompreensíveis para a Ciência^7^. Para Moscovici, essa ordem assumiria um papel de destaque e seria vista como dotada de uma atividade própria^1^. 

Assim, na lida com a verdade de seu objeto, a psicanálise empreendeu uma mudança significativa em relação à filosofia moderna ao propor a descentralização da razão e ao colocar a consciência sob suspeita, atestando a dimensão de uma subjetividade, ao mesmo tempo em que afirma a existência do desconhecido e do não sabido^7^.

Em instância diametralmente oposta ao inconsciente, as reflexões de Durkheim^6^ indicam que os fatos sociais são externos ao indivíduo e existem independentemente de nossa consciência sobre eles. Para esse autor, o ser humano passa a ter contato com esses fatos desde o nascimento e internaliza muitos deles. Moscovici foi influenciado por Durkheim na construção da TRS. Para ambos os autores, as práticas mentais têm origem na sociedade e constituem, portanto, categorias sociais de pensamento^1^ ^,^ ^6^. 

No entanto, destaca-se que, na Sociologia, há várias formas de sistematizar o entendimento sobre as representações. Os autores do pós-guerra apresentam um ponto convergente em suas obras: a superação da dicotomia entre subjetividade e objetividade na relação entre indivíduo e sociedade. Sociólogos contemporâneos a Moscovici, como o francês Pierre Bourdieu, desde o início de suas trajetórias acadêmicas enfatizaram a importância da dimensão simbólica na construção da realidade social, buscando compreender como os indivíduos constroem significados no contexto social^8^. 

Bourdieu concluiu que as representações se materializam nas práticas sociais e nas instituições, porém não são independentes delas. Para o autor, o objeto deve ser entendido a partir de uma perspectiva relacional, na qual cada elemento é definido pelas relações que o conectam a outros dentro de uma vasta teia de sistemas. Nessa teia, os elementos adquirem seus significados e funções na sociedade^8^. As diferenças entre os postulados teóricos de Moscovici e Bourdieu acerca das representações distinguem-se pela ênfase aplicada às construções simbólicas. Moscovici atribui maior destaque à estrutura estruturante; em contrapartida, para Bourdieu o relevo encontra-se na estrutura estruturada^1^ ^,^ ^8^. Ou seja, Moscovici concentra-se no processo de construção das estruturas, enquanto Bourdieu enfatiza as estruturas já estabelecidas.

A geração de Moscovici discutia intensamente como a Ciência influenciava a transformação histórica, as formas de pensamento e as perspectivas sociais, além de analisar como essas ideias poderiam ser integradas às crenças das pessoas^1^. Uma crença pode ser entendida como uma ideia sustentada por um grupo cultural específico, servindo de base para avaliações que levam à aceitação de algo como verdadeiro, mesmo sem evidências. Assim, uma representação consiste em um conjunto organizado dessas crenças, que orienta um grupo de pessoas em relação a um objeto no espaço social^1^. 

A distinção entre crença e conhecimento reside no fato de que o conhecimento científico, teoricamente, pode ser demonstrado por evidências empíricas. Moscovici indagava como o conhecimento científico se transformava em crença e conhecimento “espontâneo”^1^. O autor buscava superar o modelo científico que atribuía importância excessiva ao conhecimento científico em detrimento da cultura do senso comum, construída e moldada pela comunicação cotidiana e que constitui uma cultura. 

Sua tese versa sobre o conhecimento da sociedade francesa acerca da psicanálise, e suas conclusões podem ser sintetizadas em três aspectos fundamentais. Primeiro, as representações sociais funcionam como um ponto intermediário entre a compreensão da psicanálise pela sociedade francesa e o conhecimento científico. Segundo, as representações não possuem o mesmo significado para todos os membros da sociedade, pois dependem do contexto sociocultural dos indivíduos. Terceiro, diante de novas situações ou novos objetos, como a psicanálise, o processo de representar segue uma sequência lógica: familiarizar objetos desconhecidos por meio de um mecanismo duplo denominado ancoragem e objetivação. Esse processo permite que indivíduos ou grupos conectem imagens reais, concretas e compreensíveis, retiradas de seu cotidiano, aos novos objetos que encontram e precisam compreender^1^.

Os processos de ancoragem e objetivação são essenciais para entender como as representações sociais são formuladas, compartilhadas e transmitidas dentro de um grupo, influenciando a percepção e as respostas das pessoas ao mundo ao seu redor. O conceito de ancoragem refere-se ao processo pelo qual novas informações são relacionadas a conceitos ou ideias já existentes na mente das pessoas. Por exemplo, quando indivíduos tomam conhecimento de uma nova terapia médica, podem ancorar essa informação em suas crenças prévias sobre saúde, medicina e tratamentos. Trata-se do processo pelo qual aquilo que é estranho é incorporado a um sistema de pensamento social preexistente, permitindo que antigas representações acolham as novas, de modo que o desconhecido se torne familiar^1^.

Já o conceito de objetivação refere-se ao processo pelo qual as representações sociais são expressas e manifestadas em objetos, práticas ou símbolos concretos. Portanto, as representações sociais tornam-se visíveis e tangíveis na vida cotidiana por meio do processo de objetivação^1^. Isso pode incluir rituais, linguagem, artefatos culturais e instituições que refletem e reforçam as crenças e os valores compartilhados dentro de um grupo social. Por exemplo, símbolos da área da saúde, como jalecos brancos, estetoscópios e xamãs, podem representar crenças e valores de uma comunidade acerca do tratamento da saúde. A objetivação de um conceito ou objeto também depende de fatores socioculturais, como o acesso diferenciado à informação em função da inserção social dos indivíduos e dos valores e normas do grupo ao qual pertencem.

As representações sociais são alegóricas, situando-se entre o que pode ser observado e aquilo que não pode ser atingido diretamente. Assim, compreende-se que as representações sociais constituem realidades concretas, embora não necessariamente palpáveis ou mensuráveis. Pode-se compará-las à noção de carisma: algo impreciso, mas que, quando presente em determinadas pessoas, exerce uma influência real e concreta^1^. O carisma é uma manifestação concreta, mas praticamente intangível. As representações sociais são essa substância que se mistura com ideias e crenças, como ocorre nas religiões, nos mitos e na ciência. Elas são fenômenos específicos ligados a uma forma particular de compreender e se comunicar, fundamentais para a constituição da realidade e do senso comum, compondo a simbologia do tecido social.

## Discussão

As noções contemporâneas de saúde e Enfermagem não se constituem exclusivamente a partir de avanços técnico-científicos, mas são atravessadas por representações sociais historicamente produzidas, que orientam modos de pensar, avaliar e praticar o cuidado. Nesse sentido, os conhecimentos que sustentam determinadas epistemologias em saúde expressam crenças compartilhadas em contextos socioculturais específicos^9^. 

Conforme a TRS, esses conhecimentos não circulam de forma neutra, mas são ancorados em sistemas simbólicos preexistentes e objetivados nas práticas, nos discursos institucionais e também nos modelos de atenção existentes^1^. Assim, aquilo que se reconhece como saber válido em saúde e Enfermagem reflete não apenas critérios científicos, mas também representações sociais próprias de uma época, que influenciam a definição de normalidade, risco e cuidado^9^.

Um exemplo disso pode ser observado nas representações sociais em torno do corpo, da saúde, da doença e do risco, que, em diferentes contextos históricos, definem o que é considerado normal ou patológico^9^. Os conhecimentos científicos, ao circularem socialmente, são apropriados, reinterpretados e transformados em saberes de senso comum, orientando práticas institucionais e profissionais^1^ ^,^ ^10^. No campo da saúde, esse processo torna-se evidente quando determinados modelos de atenção, como o hospitalocêntrico ou o biomédico, se consolidam não apenas por sua eficácia técnica, mas por estarem ancorados em representações sociais que associam cuidado à intervenção especializada, à medicalização e à centralidade de um saber profissional^11^ ^-^ ^12^.

Sob essa perspectiva, as epistemologias em saúde e Enfermagem são construídas em permanente diálogo com os sistemas simbólicos de sua época, expressando valores e crenças socialmente partilhados. Nesse campo, as representações sociais desempenham papel importante na forma como as pessoas percebem, expressam e respondem aos cuidados. Elas incorporam crenças, valores, estereótipos, atitudes e comportamentos compartilhados dentro de uma comunidade ou grupo social específico^1^. Essas representações podem influenciar diversos aspectos do cuidado em saúde, como a busca por tratamento, a adesão ao tratamento prescrito, a percepção do risco de contrair doenças, as atitudes em relação aos profissionais de saúde e as escolhas de estilo de vida relacionadas à saúde. 

O grande paradoxo do estudo das representações, no âmbito da saúde, consiste em tentar explicar um fenômeno partindo da impossibilidade de conhecer a essência das coisas^12^. Destaca-se que a doença não é uma experiência limitada apenas à alteração biológica, mas é também um substrato de uma construção sociocultural. A noção de doença depende da concepção sobre o que ela representa ou significa para o ser humano^11^. Enfatizar a doença como reflexo da combinação entre aspectos da experiência individual e contextos socioculturais não implica reduzi-la a uma construção social, desconsiderando os aspectos biológicos. Pelo contrário, a discussão sociocultural deve estar articulada aos aspectos biológicos da doença, de modo a favorecer uma perspectiva interdisciplinar e integrada na abordagem desse fenômeno.

Mesmo sob o ponto de vista formal, a relação entre serviços de saúde e usuários envolve muitos outros aspectos além do encontro físico entre profissional e paciente nos ambientes onde o cuidado é prestado. Entre esses fatores, destacam-se as políticas de saúde locais e as concepções dos indivíduos sobre o que significa estar doente e o que representa socialmente procurar auxílio profissional. Dessa maneira, em cada cultura e sociedade, a doença, assim como as respostas a ela, os indivíduos que a vivenciam, aqueles que se ocupam em tratá-la e as instituições envolvidas encontram-se interconectados em uma grande teia de significados e representações^12^. 

Os componentes deste sistema incluem, entre outros elementos, as crenças sobre a origem das doenças, as formas de busca e avaliação do tratamento, os papéis desempenhados e as relações de poder entre todos os atores envolvidos^12^. Por exemplo, um resfriado específico pode ser causado pelo mesmo agente infeccioso em qualquer lugar do mundo, causando alterações fisiopatológicas semelhantes em diferentes pessoas. No entanto, o sistema de saúde disponível, os tratamentos oferecidos e a percepção que cada indivíduo acometido terá sobre sua saúde podem se manifestar de maneiras diversas.

Sob essa perspectiva, o conceito de eficácia simbólica é mobilizado para caracterizar os efeitos obtidos mediante o envolvimento das crenças e de suas simbologias no mundo físico-orgânico, mais especificamente no organismo humano, diluindo a barreira entre o consciente e o inconsciente. No mundo moderno, a cura provém muitas vezes de um concentrado bioquímico ingerido para produzir a resposta orgânica esperada. O que muitas vezes não se considera é que a crença pode atuar do inconsciente para o consciente, implicando também mudanças no *status quo* do organismo^11^. 

A interpretação da doença, assim, desloca-se para o campo da intersubjetividade, da experiência cotidiana e dos modos pelos quais os indivíduos expressam, interpretam e comunicam suas vivências. O enfrentamento do sofrimento também se vincula às estruturas sociais, e não apenas ao indivíduo. Portanto, o sofrimento relacionado à doença é uma construção social coletiva da realidade, frequentemente influenciada por contextos histórico-culturais, ideológicos e sociopolíticos de relação com a doença.

Para ilustrar essa reflexão, pode-se mencionar o papel do xamã em um parto laborioso de uma mulher indígena e as implicações dos rituais musicais e de cura nos modos como essa mulher interpreta e atribui significado à dor associada aos processos parturitivos^13^. A partir dessa reflexão, talvez se encontrem pistas para compreender o poder de cura efetivado por um placebo, bem como o papel das representações sociais ancoradas em informações proferidas por profissionais da saúde, que podem repercurtir em indicadores de morbimortalidade. Um exemplo disso é o elevado número de cesáreas eletivas realizadas no Brasil, muitas vezes sem indicação clínica e, nesses casos, sem vantagens científicas que as legitimem. 

Nessa perspectiva, determinadas representações sociais podem orientar práticas institucionais que naturalizam desigualdades, legitimam condutas inadequadas ou sustentam intervenções dissociadas do melhor conhecimento científico disponível, produzindo efeitos concretos e, por vezes, graves danos sociais. Estudos ancorados na TRS evidenciam que as práticas profissionais não são neutras, mas atravessadas por crenças, atitudes e comportamentos compartilhados no interior dos campos institucionais, que orientam decisões clínicas e organizacionais, mesmo em contextos de elevada normatividade técnica. 

Pesquisa realizada com profissionais de Enfermagem demonstra que determinadas representações estruturam modos de agir e justificar práticas assistenciais, ainda que em tensão com diretrizes científicas consolidadas^10^ ^,^ ^14^. Situações como o racismo institucional, a deslegitimação do sofrimento de determinados grupos ou a adesão a modelos terapêuticos ideologicamente orientados, como observado durante a pandemia de COVID-19, com a prescrição de medicamentos sem evidência científica^14^, ilustram como representações sociais, quando incorporadas às práticas profissionais, podem comprometer a qualidade do cuidado, tensionar princípios éticos e impactar negativamente os desfechos em saúde.

Entre os estudos sobre representações sociais encontram-se aqueles que buscam compreender a origem de determinadas representações, identificando-as por meio da linguagem, ou seja, da fala e do comportamento humano, individual ou coletivo. Nesse sentido, pesquisa^15^ realizada no aldeado francês de Ainay-le-Château, onde estava em curso uma experiência de desospitalização de pessoas com sofrimento psíquico, buscou compreender como a loucura era representada. O processo de desospitalização estimulava a convivência com moradores locais por meio de um sistema de hospedagem. Para tanto, formulou-se a seguinte pergunta norteadora: “Como as representações sociais da loucura explicam a relação com o doente mental, figura da alteridade?”. 

A pesquisa concluiu, entre outros aspectos, que os moradores predominantemente associavam o louco ou a loucura ao medo de contágio. Essa percepção levava-os a adotar comportamentos em desacordo com os conhecimentos científicos difundidos na época. Embora os moradores afirmassem, em seus discursos, que o sofrimento psíquico não era transmissível por meio do compartilhamento de utensílios domésticos e roupas, suas ações refletiam o contrário.

Dessa forma, as representações sociais continham aspectos não verbalizados que se materializavam no comportamento cotidiano dos aldeões, como lavar separadamente as roupas e utensílios das pessoas em sofrimento psíquico. Quando questionados sobre tais práticas, os moradores respondiam que se tratava de “costume”, herdado de seus pais e avós por medo de contágio. Assim, embora negassem verbalmente a possibilidade de transmissão, mantinham na prática a rígida separação de águas no cotidiano doméstico, mesmo diante da “irracionalidade” desse comportamento^15^. 

Como prática social, estudos nesse núcleo do saber têm se debruçado sobre diferentes necessidades em saúde, com apoio na TRS, permitindo avanços em diferentes temas e contextos de cuidado. A incorporação da TRS marcou uma inflexão epistemológica na produção científica da Enfermagem, ao possibilitar a superação de abordagens estritamente positivistas e ampliar a compreensão do cuidado como prática social e simbólica^2^. A TRS demonstra afinidade com os objetos da Enfermagem, que se caracterizam pela recusa à hierarquização dos saberes e pela valorização dos entrecruzamentos entre conhecimento científico, senso comum e crenças^3^. A análise do percurso das pesquisas em Enfermagem revela que esse referencial tem sido particularmente fecundo para interpretar fenômenos complexos do processo saúde-doença, da prática assistencial e da identidade profissional, permitindo compreender como crenças, atitudes e comportamentos se estabilizam e se transformam no cotidiano institucional. 

Estudo que analisou as representações sociais de enfermeiros sobre o atendimento a mulheres em situação de violência sexual revelou que tais representações se ancoram na execução de protocolos de forma humanizada, com objetivação no acolhimento^16^. Outro estudo evidenciou que, para os enfermeiros, a qualidade do cuidado em contextos hospitalares está fortemente influenciada por limitações estruturais, como superlotação e escassez de recursos materiais^17^. 

É possível verificar que as representações sociais influenciam as formas de interação com o meio social. As abordagens de pesquisa social, à luz da TRS, aplicadas à Enfermagem têm como objetivo explorar como o cuidado é percebido e entender os significados que lhe são atribuídos. Isso envolve analisar os contextos que moldam esses significados, permitindo uma melhor compreensão dos comportamentos, atitudes e crenças das pessoas em relação à saúde e à doença. Essas abordagens também auxiliam na formulação de planos terapêuticos mais alinhados às necessidades daqueles que recebem o cuidado^18^.

Nesse contexto, é importante analisar as crenças pessoais envolvidas na interação entre o indivíduo que recebe cuidados de saúde e o sistema que os oferece, a fim de subsidiar o aprimoramento de políticas, programas, serviços e da formação de profissionais de saúde. Compreender como os usuários percebem os processos de saúde e doença é fundamental para melhorar a qualidade dos serviços, aumentando o acesso e a eficácia dos cuidados. Dessa forma, torna-se necessário compreender as opiniões e os comportamentos dos usuários diante do adoecimento. Esse conhecimento pode revelar lacunas nos serviços percebidas pelos próprios usuários, influenciando a maneira como grupos similares respondem aos tratamentos e às orientações preventivas. Portanto, pesquisas acerca das representações relacionadas aos cuidados em saúde são fundamentais para orientar a formulação de políticas de saúde mais adequadas.

No entanto, destaca-se que a tentativa de definir rigidamente o conceito de representações sociais incorre no risco de transformar algo que deveria ser interpretado como situacional em algo permanente. Exemplos disso são pesquisas que procuram representar o “pensamento dos jovens”, o “pensamento das mulheres” ou as “visões de mundo de pessoas pobres”, evidenciando a necessidade de cautela na utilização desse conceito. É importante reconhecer que opiniões compartilhadas por informantes frequentemente provêm de contextos culturais heterogêneos, que nem sempre podem ser unificados por meio de enquetes^19^.

Um observador competente é aquele que não apenas observa os outros, mas também observa a si mesmo, promovendo uma interação consciente que enfatiza a alteridade. O conhecimento se constrói no confronto entre diferentes posições culturais presentes nessa interação^8^. O pesquisador não é neutro enquanto ouve e observa; ele procura entender a perspectiva do observado e confrontá-la com a sua própria. Além disso, é importante reconhecer que o investigador, na condição de intérprete, transforma- se em seu próprio leitor, e o observador torna- se um produto de sua própria reflexão^5^.

Em algumas ocasiões, o positivismo e a lógica hipotético-dedutiva sugerem, erroneamente, que há uma racionalidade neutra e que a Ciência pode se desenvolver sem estar influenciada pelas representações sociais de um contexto específico^1^. A título de exemplo, ao refletir sobre a urgência de eliminar as mortes maternas por causas evitáveis, pode-se questionar se não estariam também ancoradas em representações sociais imbricadas em concepções filosóficas sobre a morte e sociais sobre a maternidade, estruturadas por convergências históricas, políticas e econômicas que marcam a Ciência concebida em determinado espaço-tempo. 

Dirigir a atenção ao próprio ponto de vista em relação ao objeto, e a tudo o que dessa relação resulta, é condição *sine qua non* ^8^. Dessa forma, sugere-se abandonar o lugar previamente indicado e reconhecido de observador objetivo e objetivante, que impõe ao objeto suas próprias normas de construção. 

O desafio reside em evitar o realismo estrutural ao qual o objetivismo inevitavelmente conduz, tendência que atribui uma realidade absoluta a algo que é relativo - uma forma de hipostasia. Isso ocorre quando se consideram as estruturas como realidades pré-estabelecidas, independentes da história dos indivíduos e dos grupos que as compartilham. Ao mesmo tempo, é essencial não incorrer no subjetivismo, incapaz de lidar adequadamente com as complexidades do mundo social. Para superar essa dicotomia, é necessário retornar à prática - espaço da dialética no qual se encontram os produtos históricos incorporados, as macroestruturas e os campos de poder^8^.

Situar o contexto das representações sociais, buscando compreender seus mecanismos de construção, pode ser um caminho válido para analisar valores e sentidos elaborados por determinados segmentos sociais. Em consonância com essa reflexão, apresentam-se alguns questionamentos que auxiliam a evitar tais equívocos: Quem sabe e de onde sabe? O que e como sabe? Sobre o que sabe e com que efeito? Essas perguntas remetem às condições de produção e circulação das representações sociais; aos processos e estados das representações sociais; e ao estatuto epistemológico das representações sociais^15^. 

A análise sócio-histórica exige do pesquisador uma postura crítica capaz de abarcar a estrutura social, compreendendo-a como epaço de assimetrias e diferenças relativamente estáveis que caracterizam as instituições sociais e os campos de interação nas estruturas sociais. Para ilustrar essa premissa, pode-se mencionar as diferenças entre narrativas sobre a doença elaboradas por mulheres e homens, que refletem os papéis de gênero desempenhados nos grupos analisados. 

A experiência de estar doente pode compartilhar aspectos comuns entre as pessoas, independentemente do sexo biológico. No entanto, os papéis desempenhados e os significados atribuídos a essa condição podem variar significativamente, assumindo características específicas conforme as contruções culturais de gênero. Em algumas regiões do mundo, as diferenças culturais e os papéis sociais desempenhados por homens e mulheres são tão marcantes que, embora coexistam sob uma mesma bandeira, esses grupos vivem realidades quase como se pertencessem a universos distintos. Dessa forma, observa-se que os grupos sociais constroem suas próprias representações ao identificar, interpretar, aceitar ou rejeitar determinados valores e modos de viver, constituindo suas próprias significações^20^.

Este ensaio contribui para o avanço do conhecimento científico ao retomar criticamente os fundamentos epistemológicos da TRS no campo da saúde e da Enfermagem, deslocando o foco predominante da literatura, centrado na aplicabilidade empírica, para a problematização de seus pressupostos teóricos e de suas condições de uso. O estudo evidencia como as representações sociais operam não apenas na recepção das práticas de cuidado, mas também na construção dos próprios saberes, modelos de atenção e práticas profissionais em saúde. Dessa forma, o manuscrito oferece subsídios para um uso mais rigoroso, crítico e contextualizado da TRS em pesquisas futuras, contribuindo para qualificar sua incorporação teórica no campo da saúde coletiva e da Enfermagem.

Como limitações, destaca-se que, por se tratar de um ensaio teórico-reflexivo, o estudo não se propõe a realizar análise empírica nem revisão sistemática da literatura, o que restringe a possibilidade de generalizações ou de comparação direta entre diferentes contextos de aplicação da TRS. Além disso, a seleção das obras privilegiou textos clássicos e autores de referência, o que, embora coerente com o objetivo epistemológico da reflexão, pode não contemplar a totalidade das produções contemporâneas que utilizam a teoria em abordagens empíricas específicas de forma sistematizada. Ainda assim, tais limites não comprometem a validade da proposta, uma vez que possibilitaram o aprofundamento da reflexão teórica e epistemológica, sem a pretensão de esgotar o debate ou mapear a produção existente.

## Conclusão

A TRS, aplicada aos cuidados em saúde e Enfermagem, contribui para iluminar as crenças, atitudes e comportamentos que influenciam a prestação de cuidados, possibilitando uma abordagem mais eficaz e integradora no cuidado às pessoas. Compreender as representações sociais no campo da saúde é essencial para o desenvolvimento de intervenções e para o fortalecimento de práticas de cuidado mais eficazes. Isso envolve reconhecer e respeitar as crenças e valores das comunidades locais, bem como dialogar com seus membros no processo de elaboração de programas de saúde culturalmente sensíveis. 

Contudo, a aplicação da TRS também apresenta desafios epistemológicos que merecem ser explicitados. Observa-se, com frequência, que a insuficiência epistemológica se torna uma barreira para ultrapassar a instância do senso comum em pesquisas sobre representações sociais relacionadas aos cuidados em saúde e Enfermagem. Destaca-se ainda o risco de reificação das representações sociais quando estas são interpretadas como estruturas permanentes e homogêneas, desconsiderando seu caráter dinâmico e relacional. Tal interpretação pode conduzir a generalizações indevidas e à simplificação de contextos marcados por heterogeneidades sociais, históricas e culturais. 

## Data Availability

Todos os dados gerados ou analisados durante este estudo estão incluídos neste artigo publicado.

# Translation: Representaciones sociales como matriz epistemológica del cuidado en salud y Enfermería^*^

**DOI:** 10.1590/1518-8345.8047.4975

**Authors:** Lise Maria Carvalho Mendes, Nayara Gonçalves Barbosa, Thais de Oliveira Gozzo, Diene Monique Carlos, Ana Karina Bezerra Pinheiro, Flávia Azevedo Gomes-Sponholz

**Affiliations:** 1 Universidade de São Paulo, Escola de Enfermagem de Ribeirão Preto, PAHO/WHO Collaborating Centre for Nursing Research Development, Ribeirão Preto, SP, Brasil.; 2 Universidade Federal do Amapá, Departamento de Ciências Biológicas e Saúde, Macapá, AP, Brasil.; 3 Universidade de São Paulo, Escola de Enfermagem, São Paulo, SP, Brasil.; 4 Universidade Federal do Ceará, Fortaleza, CE, Brasil.; 5 Scholarship holder at the Conselho Nacional de Desenvolvimento Científico e Tecnológico (CNPq), Brasil.

**Keywords:** Representación Social, Conocimiento, Humanización de la Atención, Modelos Teóricos, Modelos Psicológicos, Psicología Social.

## Abstract

**Objetivo:**

reflexionar sobre los aspectos epistemológicos de la Teoría de las Representaciones Sociales en el campo del cuidado en salud y Enfermería.

**Método:**

se trata de un ensayo teórico-reflexivo en el cual se utilizó, como base conceptual, la lectura de los principales textos que influyeron en el desarrollo de esta teoría. Dichos textos fueron articulados con la literatura del área de la salud.

**Resultados:**

las representaciones sociales constituyen la forma en que los grupos interpretan la realidad en la que viven y orientan sus conductas y prácticas. Actúan como una sustancia simbólica situada entre aquello que puede ser observado y aquello que no puede ser alcanzado. Desde esta perspectiva, permiten comprender cómo los individuos y los grupos formulan sus creencias a partir de sus experiencias y de los valores atribuidos a sus vivencias. En las investigaciones sobre cuidado en salud y Enfermería, esta teoría busca comprender las representaciones construidas en torno al cuidado, así como conocer los significados que se le atribuyen.

**Conclusión:**

las representaciones sociales aproximan a los investigadores a los contextos que sustentan los significados, permitiendo comprender las creencias, actitudes y comportamientos de las personas relacionados con los procesos de salud y enfermedad, y contribuyendo a orientar la elaboración de planes terapéuticos más alineados con las necesidades de quienes reciben el cuidado.

## Destacados

**(1)** La Teoría de las Representaciones Sociales permite comprender el cuidado más allá de su dimensión técnica.

**(2)** Los conocimientos no circulan de manera neutra.

**(3)** La naturalización de las desigualdades en el cuidado institucionalizado es un desafío que debe superarse.

**(4)** El uso riguroso de la Teoría de las Representaciones Sociales cualifica los análisis situados y evita lecturas universalizantes.

## Introducción

La Teoría de las Representaciones Sociales (TRS), propuesta por el rumano Serge Moscovici en la década de 1960^1^, se inscribe en el universo de aquello que es aprehendido y exteriorizado por medio del discurso, pero también de aquello que no se dice y emerge en la práctica cotidiana, en el comportamiento, en la repetición, en lo colectivo y en lo individual. El individuo, al intentar apartarse de la práctica predominante de la colectividad, señala que constituye una excepción y, por lo tanto, confirma la existencia de una determinada regla. 

La TRS destaca cómo las personas comparten y construyen significados comunes sobre objetos, acontecimientos y fenómenos sociales mediante procesos de comunicación e interacción social. Estas representaciones configuran la manera en que percibimos el mundo que nos rodea e influyen en nuestras creencias, actitudes y comportamientos. Esta relación dialéctica establecida entre los aspectos individuales y sociales constituye la base teórica del concepto de la TRS, que busca comprender la apropiación de la realidad social por parte del individuo o el modo en que lo social es internalizado^1^. 

Para aportar una contribución epistémica acerca de las representaciones sociales aplicadas a los estudios sobre el cuidado en salud, se hace necesario articular el concepto de la TRS con lo que hoy se concibe como Ciencia. Se considera que este ejercicio puede actuar como un elemento conductor para comprender los avances que los estudios apoyados en este referencial pueden vislumbrar. 

Aunque la TRS ha sido ampliamente movilizada en las investigaciones en salud y Enfermería, con contribuciones relevantes ya sistematizadas en la literatura, se observa que gran parte de estos estudios ha privilegiado su aplicabilidad empírica o metodológica^2^ ^-^ ^3^. Con el tiempo, la recurrencia de estas producciones ha consolidado la TRS como un referencial fecundo para comprender los sentidos atribuidos al cuidado, sin que ello haya agotado la necesidad de reflexionar sobre sus fundamentos epistemológicos. 

En este contexto, la motivación de este ensayo surge de la necesidad de retomar críticamente los presupuestos epistemológicos de la TRS, especialmente frente a su creciente incorporación y, en ocasiones, acrítica, en el campo de la salud y la Enfermería. Más que reiterar sus contribuciones ya reconocidas, este manuscrito propone un desplazamiento reflexivo, interrogando los modos en que la TRS ha sido apropiada y los límites de su aplicación cuando se la desvincula de su matriz teórica original, con el fin de cualificar su uso crítico y contextualizado en el presente. 

De esta manera, este artículo tiene como objetivo reflexionar sobre los aspectos epistemológicos de la Teoría de las Representaciones Sociales en el campo del cuidado en salud y Enfermería.

## Método

### Diseño del estudio 

Se trata de un ensayo teórico-reflexivo^4^, entendido como una modalidad de producción científica orientada al análisis crítico, interpretativo y argumentativo de conceptos, teorías y presupuestos epistemológicos, sin la pretensión de agotamiento empírico ni de sistematización exhaustiva de la literatura. Este tipo de estudio privilegia el ejercicio reflexivo, fundamentado en marcos teóricos consistentes, articulando trayectorias conceptuales, tensiones y límites interpretativos a partir de la experiencia académica de los autores y del diálogo crítico con obras clásicas y contemporáneas^4^, con el objetivo de reflexionar sobre los aspectos epistemológicos de la TRS^1^ en el campo del cuidado en salud y Enfermería. 

### Definición de la muestra

Se realizó un relevamiento de las obras fundacionales de la TRS, con especial énfasis en los trabajos de Serge Moscovici, que delinearon los fundamentos de esta teoría en el campo de la psicología social. Asimismo, se incluyeron textos de referencia de otros autores clásicos, como Émile Durkheim y Sigmund Freud, y de autores contemporáneos, como Pierre Bourdieu, con el propósito de comprender las reflexiones teóricas y contextuales implicadas en la formulación de la TRS. 

Los textos fueron seleccionados en función de su relevancia para el análisis epistemológico de la teoría y para su aplicación en el campo del cuidado en salud. Además de las obras principales, se incluyeron estudios que exploran conceptos relacionados, como aquellos que examinan la relación entre representaciones sociales y prácticas culturales y de salud.

### Período

El recorte temporal del relevamiento y de la lectura se concentró en el período comprendido entre enero y abril de 2024, sin perjuicio de la incorporación de obras clásicas de carácter atemporal. 

### Tratamiento y análisis de los datos

La reflexión teórica se llevó a cabo mediante un análisis comprensivo e interpretativo de los textos seleccionados^5^, orientado por presupuestos de la análisis sociohistórica y epistemológica. La lectura de los textos fue guiada por una perspectiva crítica y reflexiva, priorizando: (i) la comprensión de los fundamentos epistemológicos de las obras; (ii) la identificación de convergencias, tensiones y desplazamientos conceptuales entre los autores; y (iii) la articulación de los conceptos con el campo empírico y teórico del cuidado en salud y Enfermería. 

A lo largo del proceso se realizaron tres reuniones entre los autores para discutir los textos analizados y construir consensos analíticos. Estas discusiones colectivas orientaron tanto la organización de las reflexiones como la estructura argumentativa del ensayo, permitiendo el refinamiento conceptual y la coherencia interna del texto. A partir de este recorrido analítico se organizó el eje reflexivo que orientó la presentación de los resultados, denominado “Una mirada sobre las representaciones sociales”, construido como síntesis interpretativa de los fundamentos conceptuales movilizados por los autores analizados.

### Aspectos éticos

Se trata de un ensayo teórico fundamentado exclusivamente en fuentes secundarias de dominio público, sin participación de seres humanos ni recolección de datos primarios. Por lo tanto, el estudio no se encuadra en las disposiciones de las resoluciones del Consejo Nacional de Salud de Brasil (CNS) nº 466/2012 y nº 510/2016, por lo que queda dispensada la evaluación por un Comité de Ética en Investigación.

## Resultados

Inicialmente, es posible destacar la influencia del sociólogo francés Émile Durkheim, considerado precursor de las representaciones sociales, quien en sus reflexiones presentó aportes relevantes para el estudio de las representaciones colectivas^6^. El autor enfatizó la importancia de las representaciones colectivas en la cohesión y en el mantenimiento del orden social. Argumentó que las creencias, valores y normas compartidos por los miembros de una sociedad forman una conciencia colectiva que moldea el comportamiento individual y promueve la cohesión social^6^. 

En el contexto del desarrollo histórico de las Ciencias, la estructura teórico-clínica de Sigmund Freud también ejerció influencia sobre Serge Moscovici. Este utilizó dicha influencia para formular una comprensión de las lagunas presentes en las manifestaciones de la conciencia, sugiriendo la existencia de otro orden^7^. Para Freud, el inconsciente no debe ser visto como una manifestación de irracionalidad ni como una conciencia oculta. Por el contrario, lo considera una hipótesis legítima y esencial, sin la cual los fenómenos relacionados con el funcionamiento del aparato psíquico continuarían pareciendo incoherentes e incomprensibles para la Ciencia^7^. Para Moscovici, ese orden asumiría un papel destacado y sería entendido como dotado de una actividad propia^1^. 

Así, en el tratamiento de la verdad de su objeto, el psicoanálisis emprendió un cambio significativo con respecto a la filosofía moderna al proponer la descentralización de la razón y al colocar la conciencia bajo sospecha, afirmando la dimensión de una subjetividad, al mismo tiempo que reconoce la existencia de lo desconocido y de lo no sabido^7^.

En una instancia diametralmente opuesta al inconsciente, las reflexiones de Émile Durkheim^6^ indican que los hechos sociales son externos al individuo y existen independientemente de nuestra conciencia sobre ellos. Para este autor, el ser humano entra en contacto con estos hechos desde el nacimiento e internaliza muchos de ellos. Serge Moscovici fue influenciado por Durkheim en la construcción de la TRS. Para ambos autores, las prácticas mentales tienen su origen en la sociedad y constituyen, por lo tanto, categorías sociales del pensamiento^1^ ^,^ ^6^. 

No obstante, cabe destacar que, en la Sociología, existen diversas formas de sistematizar la comprensión de las representaciones. Los autores del período de posguerra presentan un punto convergente en sus obras: la superación de la dicotomía entre subjetividad y objetividad en la relación entre individuo y sociedad. Sociólogos contemporáneos a Moscovici, como el francés Pierre Bourdieu, desde el inicio de sus trayectorias académicas enfatizaron la importancia de la dimensión simbólica en la construcción de la realidad social, buscando comprender cómo los individuos construyen significados en el contexto social^8^. 

Bourdieu concluyó que las representaciones se materializan en las prácticas sociales y en las instituciones, pero no son independientes de ellas. Para el autor, el objeto debe ser comprendido desde una perspectiva relacional, en la cual cada elemento se define por las relaciones que lo conectan con otros dentro de una vasta red de sistemas. En esta red, los elementos adquieren sus significados y funciones en la sociedad^8^. Las diferencias entre los postulados teóricos de Moscovici y Bourdieu acerca de las representaciones se distinguen por el énfasis otorgado a las construcciones simbólicas. Moscovici atribuye mayor relevancia a la estructura estructurante; en cambio, para Bourdieu el énfasis recae en la estructura estructurada^1^ ^,^ ^8^. Es decir, Moscovici se concentra en el proceso de construcción de las estructuras, mientras que Bourdieu enfatiza las estructuras ya establecidas.

La generación de Serge Moscovici discutía intensamente cómo la Ciencia influía en la transformación histórica, en las formas de pensamiento y en las perspectivas sociales, además de analizar cómo estas ideas podían integrarse en las creencias de las personas^1^. Una creencia puede entenderse como una idea sostenida por un grupo cultural específico, que sirve de base para evaluaciones que conducen a aceptar algo como verdadero, incluso sin evidencias. De este modo, una representación consiste en un conjunto organizado de estas creencias, que orienta a un grupo de personas con relación a un objeto en el espacio social^1^. 

La distinción entre creencia y conocimiento radica en el hecho de que el conocimiento científico, en teoría, puede ser demostrado mediante evidencias empíricas. Moscovici se preguntaba cómo el conocimiento científico se transformaba en creencia y en conocimiento “espontáneo”^1^. El autor buscaba superar el modelo científico que atribuía una importancia excesiva al conocimiento científico en detrimento de la cultura del sentido común, construida y moldeada por la comunicación cotidiana y que constituye, en sí misma, una cultura. 

Su tesis versa sobre el conocimiento de la sociedad francesa acerca del psicoanálisis, y sus conclusiones pueden sintetizarse en tres aspectos fundamentales. En primer lugar, las representaciones sociales funcionan como un punto intermedio entre la comprensión del psicoanálisis por parte de la sociedad francesa y el conocimiento científico. En segundo lugar, las representaciones no poseen el mismo significado para todos los miembros de la sociedad, ya que dependen del contexto sociocultural de los individuos. En tercer lugar, frente a nuevas situaciones u objetos, como el psicoanálisis, el proceso de representar sigue una secuencia lógica: familiarizar los objetos desconocidos mediante un mecanismo doble denominado anclaje y objetivación. Este proceso permite que individuos o grupos conecten imágenes reales, concretas y comprensibles, extraídas de su vida cotidiana, con los nuevos objetos que encuentran y necesitan comprender^1^.

Los procesos de anclaje y objetivación son esenciales para comprender cómo las representaciones sociales se formulan, comparten y transmiten dentro de un grupo, influyendo en la percepción y en las respuestas de las personas frente al mundo que las rodea. El concepto de anclaje se refiere al proceso mediante el cual nuevas informaciones se relacionan con conceptos o ideas ya existentes en la mente de las personas. Por ejemplo, cuando los individuos toman conocimiento de una nueva terapia médica, pueden anclar esa información en sus creencias previas sobre salud, medicina y tratamientos. Se trata del proceso por el cual aquello que resulta extraño se incorpora a un sistema de pensamiento social preexistente, permitiendo que antiguas representaciones acojan las nuevas, de modo que lo desconocido se vuelva familiar^1^.

Por su parte, el concepto de objetivación se refiere al proceso mediante el cual las representaciones sociales se expresan y se manifiestan en objetos, prácticas o símbolos concretos. De este modo, las representaciones sociales se vuelven visibles y tangibles en la vida cotidiana por medio del proceso de objetivación^1^. Esto puede incluir rituales, lenguaje, artefactos culturales e instituciones que reflejan y refuerzan las creencias y los valores compartidos dentro de un grupo social. Por ejemplo, símbolos del campo de la salud, como las batas blancas, los estetoscopios o incluso los chamanes, pueden representar creencias y valores de una comunidad acerca del tratamiento de la salud. La objetivación de un concepto u objeto también depende de factores socioculturales, como el acceso diferenciado a la información según la inserción social de los individuos y los valores y normas del grupo al que pertenecen.

Las representaciones sociales son alegóricas y se sitúan entre aquello que puede ser observado y aquello que no puede ser alcanzado directamente. Así, se comprende que las representaciones sociales constituyen realidades concretas, aunque no necesariamente palpables o mensurables. Pueden compararse con la noción de carisma: algo impreciso, pero que, cuando está presente en determinadas personas, ejerce una influencia real y concreta^1^. El carisma es una manifestación concreta, pero prácticamente intangible. Las representaciones sociales son esa sustancia que se mezcla con ideas y creencias, como ocurre en las religiones, los mitos y la ciencia. Son fenómenos específicos vinculados a una forma particular de comprender y comunicarse, fundamentales para la constitución de la realidad y del sentido común, componiendo la simbología del tejido social.

## Discusión

Las nociones contemporáneas de salud y Enfermería no se constituyen exclusivamente a partir de avances técnico-científicos, sino que están atravesadas por representaciones sociales históricamente producidas, que orientan modos de pensar, evaluar y practicar el cuidado. En este sentido, los conocimientos que sustentan determinadas epistemologías en salud expresan creencias compartidas en contextos socioculturales específicos^9^. 

De acuerdo con la TRS, estos conocimientos no circulan de manera neutra, sino que se encuentran anclados en sistemas simbólicos preexistentes y objetivados en las prácticas, en los discursos institucionales y también en los modelos de atención existentes^1^. Así, aquello que se reconoce como saber válido en salud y Enfermería refleja no solamente criterios científicos, sino también representaciones sociales propias de una época, que influyen en la definición de normalidad, riesgo y cuidado^9^.

 Un ejemplo de ello puede observarse en las representaciones sociales en torno al cuerpo, la salud, la enfermedad y el riesgo, que, en diferentes contextos históricos, definen lo que se considera normal o patológico^9^. Los conocimientos científicos, al circular socialmente, son apropiados, reinterpretados y transformados en saberes de sentido común, orientando prácticas institucionales y profesionales^1^ ^,^ ^10^. En el campo de la salud, este proceso se vuelve evidente cuando determinados modelos de atención, como el hospitalocéntrico o el biomédico, se consolidan no solamente por su eficacia técnica, sino también por estar anclados en representaciones sociales que asocian el cuidado con la intervención especializada, la medicalización y la centralidad de un saber profesional^11^ ^-^ ^12^.

Desde esta perspectiva, las epistemologías en salud y Enfermería se construyen en permanente diálogo con los sistemas simbólicos de su época, expresando valores y creencias socialmente compartidos. En este campo, las representaciones sociales desempeñan un papel importante en la forma en que las personas perciben, expresan y responden a los cuidados. Estas incorporan creencias, valores, estereotipos, actitudes y comportamientos compartidos dentro de una comunidad o grupo social específico^1^. Dichas representaciones pueden influir en diversos aspectos del cuidado en salud, como la búsqueda de tratamiento, la adherencia al tratamiento prescrito, la percepción del riesgo de contraer enfermedades, las actitudes frente a los profesionales de la salud y las elecciones de estilo de vida relacionadas con la salud. 

La gran paradoja del estudio de las representaciones en el ámbito de la salud consiste en intentar explicar un fenómeno partiendo de la imposibilidad de conocer la esencia de las cosas^12^. Cabe destacar que la enfermedad no es una experiencia limitada únicamente a una alteración biológica, sino que también constituye el sustrato de una construcción sociocultural. La noción de enfermedad depende de la concepción acerca de lo que esta representa o significa para el ser humano^11^. Enfatizar la enfermedad como reflejo de la combinación entre aspectos de la experiencia individual y contextos socioculturales no implica considerarla únicamente como una construcción social, desestimando los aspectos biológicos. Por el contrario, la discusión sociocultural debe articularse con los aspectos biológicos de la enfermedad, de modo que se favorezca una perspectiva interdisciplinaria e integrada en el abordaje de este fenómeno.

Incluso desde un punto de vista formal, la relación entre los servicios de salud y los usuarios involucra muchos otros aspectos además del encuentro físico entre el profesional y el paciente en los entornos donde se presta el cuidado. Entre estos factores se destacan las políticas de salud locales y las concepciones de los individuos acerca de lo que significa estar enfermo y de lo que representa socialmente buscar ayuda profesional. De este modo, en cada cultura y sociedad, la enfermedad, así como las respuestas frente a ella, los individuos que la experimentan, quienes se ocupan de tratarla y las instituciones involucradas se encuentran interconectados en una gran red de significados y representaciones^12^. 

Los componentes de este sistema incluyen, entre otros elementos, las creencias sobre el origen de las enfermedades, las formas de búsqueda y evaluación del tratamiento, los roles desempeñados y las relaciones de poder entre todos los actores involucrados^12^. Por ejemplo, un resfriado específico puede ser causado por el mismo agente infeccioso en cualquier lugar del mundo, produciendo alteraciones fisiopatológicas semejantes en distintas personas. Sin embargo, el sistema de salud disponible, los tratamientos ofrecidos y la percepción que cada individuo afectado tendrá sobre su propia salud pueden manifestarse de maneras diversas.

Desde esta perspectiva, el concepto de eficacia simbólica se moviliza para caracterizar los efectos obtenidos mediante la participación de las creencias y de sus simbologías en el mundo físico-orgánico, más específicamente en el organismo humano, diluyendo la barrera entre lo consciente y lo inconsciente. En el mundo moderno, la curación proviene muchas veces de un concentrado bioquímico ingerido para producir la respuesta orgánica esperada. Lo que con frecuencia no se considera es que la creencia puede actuar desde el inconsciente hacia el consciente, implicando también cambios en el *status quo* del organismo^11^. 

La interpretación de la enfermedad, así, se desplaza hacia el campo de la intersubjetividad, de la experiencia cotidiana y de los modos mediante los cuales los individuos expresan, interpretan y comunican sus vivencias. El afrontamiento del sufrimiento también se vincula con las estructuras sociales, y no únicamente con el individuo. Por lo tanto, el sufrimiento relacionado con la enfermedad constituye una construcción social colectiva de la realidad, frecuentemente influida por contextos histórico-culturales, ideológicos y sociopolíticos de relación con la enfermedad.

Para ilustrar esta reflexión, puede mencionarse el papel del chamán en un parto laborioso de una mujer indígena y las implicaciones de los rituales musicales y de curación en los modos en que esta mujer interpreta y atribuye significado al dolor asociado a los procesos de parto^13^. A partir de esta reflexión, tal vez se encuentren pistas para comprender el poder de curación producido por un placebo, así como el papel de las representaciones sociales ancladas en informaciones transmitidas por profesionales de la salud, que pueden repercutir en indicadores de morbimortalidad. Un ejemplo de ello es el elevado número de cesáreas electivas realizadas en Brasil, muchas veces sin indicación clínica y, en esos casos, sin ventajas científicas que las legitimen. 

Desde esta perspectiva, determinadas representaciones sociales pueden orientar prácticas institucionales que naturalizan desigualdades, legitiman conductas inadecuadas o sostienen intervenciones disociadas del mejor conocimiento científico disponible, produciendo efectos concretos y, en ocasiones, graves daños sociales. Estudios anclados en la TRS evidencian que las prácticas profesionales no son neutras, sino que están atravesadas por creencias, actitudes y comportamientos compartidos en el interior de los campos institucionales, que orientan decisiones clínicas y organizacionales incluso en contextos de elevada normatividad técnica. 

Investigaciones realizadas con profesionales de Enfermería demuestran que determinadas representaciones estructuran modos de actuar y de justificar prácticas asistenciales, incluso cuando se encuentran en tensión con directrices científicas consolidadas^10^ ^,^ ^14^. Situaciones como el racismo institucional, la deslegitimación del sufrimiento de determinados grupos o la adhesión a modelos terapéuticos ideológicamente orientados, como se observó durante la pandemia de COVID-19, con la prescripción de medicamentos sin evidencia científica^14^, ilustran cómo las representaciones sociales, cuando se incorporan a las prácticas profesionales, pueden comprometer la calidad del cuidado, tensionar principios éticos e impactar negativamente en los resultados en salud.

Entre los estudios sobre representaciones sociales se encuentran aquellos que buscan comprender el origen de determinadas representaciones, identificándolas por medio del lenguaje, es decir, de la expresión verbal y del comportamiento humano, individual o colectivo. En este sentido, una investigación^15^ realizada en la localidad francesa de Ainay-le-Château, donde se desarrollaba una experiencia de deshospitalización de personas con sufrimiento psíquico, buscó comprender cómo era representada la locura. El proceso de deshospitalización estimulaba la convivencia con los residentes locales mediante un sistema de hospedaje. Para ello, se formuló la siguiente pregunta orientadora: “¿Cómo las representaciones sociales de la locura explican la relación con el enfermo mental, figura de la alteridad?”. 

La investigación concluyó, entre otros aspectos, que los habitantes asociaban predominantemente al loco o a la locura con el miedo al contagio. Esta percepción los llevaba a adoptar comportamientos en desacuerdo con los conocimientos científicos difundidos en aquella época. Aunque los habitantes afirmaban, en sus discursos, que el sufrimiento psíquico no era transmisible mediante el intercambio de utensilios domésticos y ropa, sus acciones reflejaban lo contrario.

De este modo, las representaciones sociales contenían aspectos no verbalizados que se materializaban en el comportamiento cotidiano de los aldeanos, como lavar por separado la ropa y los utensilios de las personas con sufrimiento psíquico. Cuando se les preguntaba sobre tales prácticas, los residentes respondían que se trataba de una “costumbre”, heredada de sus padres y abuelos por miedo al contagio. Así, aunque negaban verbalmente la posibilidad de transmisión, mantenían en la práctica una estricta separación de aguas en la vida doméstica cotidiana, incluso frente a la “irracionalidad” de dicho comportamiento^15^. 

Como práctica social, los estudios en este núcleo de conocimiento se han dedicado a analizar diversas necesidades en salud con el apoyo de la TRS, permitiendo avances en diferentes temas y contextos de cuidado. La incorporación de la TRS marcó una inflexión epistemológica en la producción científica de la Enfermería, al posibilitar la superación de enfoques estrictamente positivistas y ampliar la comprensión del cuidado como una práctica social y simbólica^2^. La TRS demuestra afinidad con los objetos de la Enfermería, caracterizados por el rechazo a la jerarquización de los saberes y por la valorización de los entrecruzamientos entre conocimiento científico, sentido común y creencias^3^. El análisis de la trayectoria de las investigaciones en Enfermería revela que este marco teórico ha sido particularmente fecundo para interpretar fenómenos complejos del proceso salud-enfermedad, de la práctica asistencial y de la identidad profesional, permitiendo comprender cómo las creencias, actitudes y comportamientos se estabilizan y se transforman en el cotidiano institucional. 

Un estudio que analizó las representaciones sociales de enfermeros sobre la atención a mujeres en situación de violencia sexual reveló que dichas representaciones se anclan en la ejecución de protocolos de manera humanizada, con objetivación en la acogida^16^. Otro estudio evidenció que, para los enfermeros, la calidad del cuidado en contextos hospitalarios está fuertemente influida por limitaciones estructurales, como la sobreocupación y la escasez de recursos materiales^17^. 

Es posible verificar que las representaciones sociales influyen en las formas de interacción con el entorno social. Los enfoques de investigación social, a la luz de la TRS, aplicados a la Enfermería, tienen como objetivo explorar cómo se percibe el cuidado y comprender los significados que se le atribuyen. Esto implica analizar los contextos que moldean dichos significados, permitiendo una mejor comprensión de los comportamientos, actitudes y creencias de las personas con relación a la salud y la enfermedad. Estos enfoques también contribuyen a la formulación de planes terapéuticos más alineados con las necesidades de quienes reciben el cuidado^18^.

En este contexto, resulta importante analizar las creencias personales implicadas en la interacción entre el individuo que recibe cuidados de salud y el sistema que los ofrece, con el fin de subsidiar el perfeccionamiento de políticas, programas, servicios y de la formación de profesionales de la salud. Comprender cómo los usuarios perciben los procesos de salud y enfermedad es fundamental para mejorar la calidad de los servicios, aumentando el acceso y la eficacia de los cuidados. De este modo, se vuelve necesario comprender las opiniones y los comportamientos de los usuarios frente al proceso de enfermedad. Este conocimiento puede revelar vacíos en los servicios percibidos por los propios usuarios, influyendo en la manera en que grupos similares responden a los tratamientos y a las orientaciones preventivas. Por lo tanto, las investigaciones acerca de las representaciones relacionadas con los cuidados en salud son fundamentales para orientar la formulación de políticas de salud más adecuadas.

No obstante, se destaca que el intento de definir rígidamente el concepto de representaciones sociales implica el riesgo de transformar algo que debería interpretarse como situacional en algo permanente. Ejemplos de ello son investigaciones que buscan representar el “pensamiento de los jóvenes”, el “pensamiento de las mujeres” o las “visiones del mundo de las personas pobres”, lo que evidencia la necesidad de cautela en el uso de este concepto. Es importante reconocer que las opiniones compartidas por los informantes provienen con frecuencia de contextos culturales heterogéneos, que no siempre pueden ser unificados mediante encuestas^19^.

Un observador competente es aquel que no solamente observa a los demás, sino que también se observa a sí mismo, promoviendo una interacción consciente que enfatiza la alteridad. El conocimiento se construye en el enfrentamiento entre diferentes posiciones culturales presentes en esa interacción^8^. El investigador no es neutral mientras escucha y observa; procura comprender la perspectiva del observado y confrontarla con la propia. Además, es importante reconocer que el investigador, en su condición de intérprete, se transforma en su propio lector, y el observador se convierte en un producto de su propia reflexión^5^.

En algunas ocasiones, el positivismo y la lógica hipotético-deductiva sugieren, erróneamente, que existe una racionalidad neutral y que la ciencia puede desarrollarse sin estar influida por las representaciones sociales de un contexto específico^1^. A modo de ejemplo, al reflexionar sobre la urgencia de eliminar las muertes maternas por causas evitables, cabe preguntarse si dichas preocupaciones no estarían también ancladas en representaciones sociales imbricadas en concepciones filosóficas sobre la muerte y sociales sobre la maternidad, estructuradas por convergencias históricas, políticas y económicas que marcan la Ciencia concebida en un determinado espacio-tiempo. 

Dirigir la atención hacia el propio punto de vista con relación al objeto, y hacia todo lo que resulta de esa relación, es condición *sine qua non* ^8^. De este modo, se sugiere abandonar el lugar previamente indicado y reconocido de observador objetivo y objetivante, que impone al objeto sus propias normas de construcción. 

El desafío consiste en evitar el realismo estructural al que el objetivismo conduce inevitablemente, una tendencia que atribuye una realidad absoluta a algo que es relativo - una forma de hipóstasis. Esto ocurre cuando las estructuras se consideran realidades preestablecidas, independientes de la historia de los individuos y de los grupos que las comparten. Al mismo tiempo, es esencial no incurrir en el subjetivismo, incapaz de abordar adecuadamente las complejidades del mundo social. Para superar esta dicotomía, es necesario retornar a la práctica - el espacio de la dialéctica en el que se encuentran los productos históricos incorporados, las macroestructuras y los campos de poder^8^.

Situar el contexto de las representaciones sociales, buscando comprender sus mecanismos de construcción, puede constituir un camino válido para analizar los valores y significados elaborados por determinados segmentos sociales. En consonancia con esta reflexión, se presentan algunos interrogantes que ayudan a evitar tales equívocos: ¿Quién sabe y desde dónde sabe? ¿Qué sabe y cómo lo sabe? ¿Sobre qué sabe y con qué efectos? Estas preguntas remiten a las condiciones de producción y circulación de las representaciones sociales; a los procesos y estados de las representaciones sociales; y al estatuto epistemológico de las representaciones sociales^15^. 

El análisis sociohistórico exige del investigador una postura crítica capaz de abarcar la estructura social, comprendiéndola como un espacio de asimetrías y diferencias relativamente estables que caracterizan a las instituciones sociales y a los campos de interacción en las estructuras sociales. Para ilustrar esta premisa, pueden mencionarse las diferencias entre las narrativas sobre la enfermedad elaboradas por mujeres y hombres, que reflejan los roles de género desempeñados en los grupos analizados. 

La experiencia de estar enfermo puede compartir aspectos comunes entre las personas, independientemente del sexo biológico. No obstante, los roles desempeñados y los significados atribuidos a esta condición pueden variar significativamente, asumiendo características específicas según las construcciones culturales de género. En algunas regiones del mundo, las diferencias culturales y los roles sociales desempeñados por hombres y mujeres son tan marcados que, aunque coexistan bajo una misma bandera, estos grupos viven realidades casi como si pertenecieran a universos distintos. De este modo, se observa que los grupos sociales construyen sus propias representaciones al identificar, interpretar, aceptar o rechazar determinados valores y modos de vida, constituyendo sus propios significados^20^.

Este ensayo contribuye al avance del conocimiento científico al retomar críticamente los fundamentos epistemológicos de la TRS en el campo de la salud y de la Enfermería, desplazando el foco predominante de la literatura, centrado en la aplicabilidad empírica, hacia la problematización de sus presupuestos teóricos y de sus condiciones de uso. El estudio evidencia cómo las representaciones sociales operan no solamente en la recepción de las prácticas de cuidado, sino también en la construcción de los propios saberes, modelos de atención y prácticas profesionales en salud. De esta manera, el manuscrito ofrece insumos para un uso más riguroso, crítico y contextualizado de la TRS en investigaciones futuras, contribuyendo a cualificar su incorporación teórica en el campo de la salud colectiva y de la Enfermería.

Como limitaciones, se destaca que, al tratarse de un ensayo teórico-reflexivo, el estudio no se propone realizar un análisis empírico ni una revisión sistemática de la literatura, lo que restringe la posibilidad de generalizaciones o de comparación directa entre distintos contextos de aplicación de la TRS. Además, la selección de las obras privilegió textos clásicos y autores de referencia, lo que, aunque resulta coherente con el objetivo epistemológico de la reflexión, puede no contemplar la totalidad de las producciones contemporáneas que utilizan la teoría en abordajes empíricos específicos de manera sistematizada. Aun así, tales límites no comprometen la validez de la propuesta, ya que permitieron profundizar en la reflexión teórica y epistemológica, sin la pretensión de agotar el debate ni de mapear la producción existente.

## Conclusión

La TRS, aplicada a los cuidados en salud y Enfermería, contribuye a iluminar las creencias, actitudes y comportamientos que influyen en la prestación del cuidado, posibilitando un enfoque más eficaz e integrador en la atención a las personas. Comprender las representaciones sociales en el campo de la salud es esencial para el desarrollo de intervenciones y para el fortalecimiento de prácticas de cuidado más eficaces. Esto implica reconocer y respetar las creencias y los valores de las comunidades locales, así como dialogar con sus miembros en el proceso de elaboración de programas de salud culturalmente sensibles. 

No obstante, la aplicación de la TRS también presenta desafíos epistemológicos que merecen ser explicitados. Se observa con frecuencia que la insuficiencia epistemológica se convierte en una barrera para trascender la instancia del sentido común en las investigaciones sobre representaciones sociales relacionadas con los cuidados en salud y Enfermería. Asimismo, se destaca el riesgo de reificación de las representaciones sociales cuando estas son interpretadas como estructuras permanentes y homogéneas, desconsiderando su carácter dinámico y relacional. Tal interpretación puede conducir a generalizaciones indebidas y a la simplificación de contextos marcados por heterogeneidades sociales, históricas y culturales. 

## Data Availability

Todos los datos generados o analizados durante este estudio están incluidos en este artículo publicado.
